# Gonad-Specific Transcriptomes Reveal Differential Expression of Gene and miRNA Between Male and Female of the Discus Fish (*Symphysodon aequifasciatus*)

**DOI:** 10.3389/fphys.2020.00754

**Published:** 2020-08-11

**Authors:** Yuanshuai Fu, Zhe Xu, Bin Wen, Jianzhong Gao, Zaizhong Chen

**Affiliations:** ^1^Key Laboratory of Freshwater Aquatic Genetic Resources, Ministry of Agriculture, Shanghai Ocean University, Shanghai, China; ^2^Key Laboratory of Exploration and Utilization of Aquatic Genetic Resources, Ministry of Education, Shanghai Ocean University, Shanghai, China; ^3^Shanghai Collaborative Innovation for Aquatic Animal Genetics and Breeding, Shanghai Ocean University, Shanghai, China

**Keywords:** *Symphysodon aequifasciatus*, ovary, testis, transcriptome, mRNAs, miRNAs

## Abstract

The discus fish (*Symphysodon aequifasciatus*) is an ornamental fish that is well-known around the world. In artificial reproduction, they must be matched by one male and one female, whereas phenotype investigation indicated that there are no significant differences in appearance between males and females, which causes great difficulties in the mating during artificial reproduction. So, it is of great importance to establish artificial sex identification methods for the discus fish. The molecular mechanism of the sexual dimorphism of the discus fish was previously unknown. In this study, we constructed six cDNA libraries from three adult testes and three adult ovaries and performed RNA sequencing for identifying sex-biased candidate genes and microRNAs (miRNAs). A total of 50,082 non-redundant genes (unigenes) were identified, of which 18,570 unigenes were significantly overexpressed in testes, and 11,182 unigenes were significantly overexpressed in ovaries. A total of 551 miRNAs were identified, of which 47 miRNAs were differentially expressed between testes and ovaries. Eight differentially expressed unigenes, seven differentially expressed miRNAs and one non-differential miRNA were validated by quantitative real-time polymerase chain reaction. Twenty-four of these differentially expressed miRNAs and their 15 predicted target genes constituted 41 miRNA–mRNA interaction pairs, and some of vital sex-related metabolic pathways were also identified. These results revealed these differentially expressed genes and miRNAs between ovary and testis might be involved in regulating gonadal development, sex determination, gametogenesis, and physiological function maintenance, and there are complex regulatory networks between genes and miRNAs. It can help us understand the molecular mechanism of the sexual dimorphism and obtain a high-efficiency sex identification method in the artificial reproduction process of the discus fish.

## Introduction

The discus fish (*Symphysodon aequifasciatus*), one of the most demanded freshwater ornamental fish species around the world, has been widely cultured because of its brilliant colors and pretty disk-shaped body. With the improvement of people’s living standard, the demand for the discus fish is increasing. Before artificial reproduction, the discus fish must be matched by one male and one female. However, the male and female of discus fish cannot be judged according to the external features, which causes great difficulties in the mating between males and females in the artificial reproduction process. As a result, the rapid reproduction of the discus fish is suppressed; its production is limited. Therefore, it is of great importance to establish artificial sex identification techniques that can be used in quick pairing of one male and one female for producing more and more progeny. A comprehensive understanding of developmental mechanism of the sexual dimorphism of the discus fish is urgently needed, including knowledge of the genes and microRNAs (miRNAs) involved in the gonads of both sexes.

In fish, there is a higher variety of sex determination mechanisms, including genetic sex determination, environmental sex determination, or even interactions of genetic and environmental sex determination (Baroiller and Guiguen, 2001; Nagahama, 2005; Baroiller et al., 2009; Shen and Wang, 2014). Gonad is an indispensable reproductive organ including testis and ovary; their development is controlled by many genes, which are differentially expressed between testes and ovaries. To date, several master sex-determining genes have been identified and play a key role in regulating sex development as transcription factors. *SRY* in mammals (Sinclair et al., 1990) and *DMY/dmrt1bY* in medaka (Matsuda et al., 2002; Nanda et al., 2002) both initiate male sex determination, are required for testis formation in XY embryos, and are sufficient to induce testis differentiation in XX embryos (Huang et al., 2017). In females, there exist a number of essential ovary-specific genes, for example, β*-catenin*, *follistatin*, *FOXL2*, *R-spondin*, and *WNT4* (Huang et al., 2017). Deficiency of these genes may cause ovarian development stasis, and mutation of these genes may result in aberrant ovary development. Gametogenesis can be differentiated in spermatogenesis and oogenesis; there are numerous gene expressions associated with gametogenesis in the reproductive stage in the mature gonads of teleost (Cabas et al., 2013). Previous studies have indicated that several key genes associated with steroid hormone enzymes are expressed during the stage of gametogenesis in tilapia (Senthilkumaran et al., 2009).

MicroRNAs are endogenously expressed, small non-coding RNAs that are approximately 18 to 22 nucleotides (nt) long and that posttranscriptionally regulate gene expression, inhibit targeted gene translation, or degrade target mRNA by partial or complete complementarity binding to the 3′ untranslated region of target genes in both animals and plants (Ambros, 2004; Bartel, 2009). The total set of transcripts (mRNA and non-coding RNA) involved in the transcriptome is transcribed at a specific organization during a particular developmental stage (Mardis, 2008). In organism, one miRNA may control the expression of several genes, or the expression of a single gene requires multiple miRNAs to work simultaneously (Bartel, 2004). Previous studies showed that miRNA may be an inducible factor to increase the complexity of organism with their roles in regulating gene expression (Sempere et al., 2006; Niwa and Slack, 2007; Heimberg et al., 2008). In miRBase Release 22.1^1^, there are the identified miRNA information from 16 teleost fish; four of them belong to the cichlid such as *Astatotilapia burtoni* (298 precursors, 236 mature), *Neolamprologus brichardi* (251 precursors, 182 mature), *Oreochromis niloticus* (812 precursors, 695 mature), and *Pundamilia nyererei* (250 precursors, 182 mature) (Brawand et al., 2014; Xiong et al., 2019). In tilapia gonads, 111 differentially expressed miRNAs (DEMs) were identified between testes and ovaries, and the targets of these sex-biased miRNAs contained key genes encoding enzymes in steroid hormone biosynthesis pathways (Tao et al., 2016). In rainbow trout, a total of 13 differential expression miRNAs were observed during stages of oogenesis, which indicated that they might regulate female gamete formation during oogenesis (Juanchich et al., 2013). There is no available information on miRNAs in the discus fish, so we have carried out preliminary research work about gonad miRNAs and release it in bioRxiv as a preprint (Fu et al., 2018).

In this study, we are aiming to screen differentially expressed genes (DEGs) and miRNAs between testes and ovaries through RNA sequencing and identify key genes and miRNAs capable of regulating gonadal development or sex determination. This work will help to further understand the underlying molecular mechanisms of sex differentiation and sex determination in the discus fish.

## Materials and Methods

### Sample Collection

Three 1-year-old healthy males (testicular development completed and visible sperm, average size 18.4 cm, average weight 158.8 g, *n* = 3) and three 1-year-old healthy females (ovarian development completed and visible eggs, average size 17.1 cm, average weight 146.9 g, *n* = 3), were obtained from the Ornamental Aquatic Breeding Laboratory in the College of Fisheries and Life Science of Shanghai Ocean University (Shanghai, China) and maintained in one feed cylinder at 28°C ± 0.5° and fed with beef heart. After 3 days, these six experimental fish were anesthetized in well-aerated water containing a 100 mg/L concentration of MS-222 (3-aminobenzoic acid ethyl ester methane sulfonate; Argent Chemical Laboratories, Redmond, WA, United States) and were killed by decapitation, and then ovaries and testes were collected by dissecting these fish and immersed into liquid nitrogen and finally stored at −80°C until subsequent RNA isolation. Our study was performed in strict accordance with Laboratory Animal–Guideline for Ethical Review of Animal Welfare of China (GB/T 35892-2018). All experimental procedures were approved by the Animal ethics committee of Shanghai Ocean University (SHOU-DW-2017-039).

### RNA Isolation

Total RNA was extracted using the miRNeasy Kit (QIAGEN, Hilden, Germany) according to the manufacturer’s protocol and treated with RNase-free DNase I (TIANGEN, Beijing, China) to remove genomic DNA contamination. The total RNA quantity and purity were analyzed by Bioanalyzer 2100 (Agilent, Technologies, Santa Clara, CA, United States) with RNA integrity number >7.0. The concentration and quality of the purified RNA samples were determined utilizing a NanoDrop 2000C spectrophotometer (Thermo Scientific, Waltham, MA, United States) and RNA integrity was detected by agarose-gel electrophoresis, and A260/A280 ratios were between 2.0 and 1.9.

### Library Construction and Sequencing for mRNA

Six gonadal samples for mRNA transcriptome analysis were prepared using a TruSeq^TM^ RNA Sample Prep Kit (Illumina, San Diego, CA, United States) according to manufacturer instructions. These mRNAs were isolated from >5 μg of gonadal total RNAs using oligo (dT) magnetic beads. These short fragment RNAs were transcribed to create first-strand cDNAs using random hexamer primers; the second-strand cDNAs were then synthesized using RNase H, buffer, dNTP, and DNA polymerase I. These double-stranded cDNAs were purified using Takara’s polymerase chain reaction (PCR) extraction kit (Takara Bio, Dalian, Liaoning, China), then ligated with sequencing adapters, and resolved by agarose gel electrophoresis. Proper fragments were selected and purified and subsequently amplified by 15 cycles of PCR to create the cDNA libraries. The DSN kit (Evrogen, Moscow, Russia) was used to normalize the cDNA libraries. These normalized cDNA libraries were sequenced on an Illumina HiSeq 2500 sequencing platform, with 125-nt reads length and both end sequencing pattern.

### Library Construction and Sequencing for Small RNA

Six gonadal samples for miRNA transcriptome analysis were prepared using a TruSeq^TM^ Small RNA Sample Prep Kit (Illumina) according to manufacturer instructions. Small RNA was isolated from gonadal total RNA and was ligated with proprietary 5′ and 3′ adapter. Adaptor-ligated small RNAs were then reverse transcribed to create cDNA constructs using Superscript reverse transcriptase (Invitrogen, Carlsbad, CA, United States). These generated small cDNA libraries were amplified by 15 cycles of PCR using Illumina small RNA primer set and Phusion polymerase (New England Lab, United States) and purified on a 6% Novex TBE PAGE gel. The purified PCR libraries were sequenced on an Illumina HiSeq 2500 sequencing platform, with 50 sequencing cycle number, 50-nt reads length, and single end sequencing pattern.

### Bioinformatics Analysis of mRNA and miRNA Transcriptome Data

The clean reads for mRNA transcriptome were obtained using NGS QC TOOLKIT v2.3.3 software (Patel and Jain, 2012) by filtering out adapter sequences, low-quality reads (reads with ambiguous bases “N”), and reads with more than 10% *Q* < 25 bases. The clean reads were assembled into non-redundant transcripts using Trinity program^2^ (Grabherr et al., 2011) with default K-mers = 25. The non-redundant transcripts less than 100 bp in length and partially overlapping sequence were removed. To analyze the conservation of the non-redundant transcripts between species, we compared all these transcripts with the NR database^3^ using DIAMOND software. Next, the remained non-redundant transcripts were annotated by Blast search against the NR protein, the Gene Ontology (GO), Clusters of Orthologous Group (COG), and Kyoto Encyclopedia of Genes and Genomes (KEGG) database using an *E* value cutoff of 10^–5^. The functional annotation by GO terms (Ashburner et al., 2000) was carried out using Blast2GO software; the functional annotation against the COG (Tatusov et al., 2003) and KEGG database (Kanehisa et al., 2008) was performed using Blast software.

The raw reads from miRNA transcriptome were subjected to initially filter and remove low-quality reads (including reads shorter than 18 nt) and adapter sequences. The clean reads with a length of ∼18 to 26 nt were subsequently aligned to Rfam 11.0 and the NCBI database searching, and these reads similar to rRNA, tRNA, snRNA, snoRNA, and scRNA were removed. The remnant reads were aligned against correlation sequences in miRBase 21 (Kozomara and Griffiths-Jones, 2014) and the reference genome of *S. aequifasciatus* non-redundant transcripts of this study, allowing length variation at both 3′ and 5′ ends and one mismatch inside of the sequence (Fu et al., 2011). The unmapped sequences were BLASTed against the specific genomes, and the hairpin RNA structures containing sequences were predicated from the flank 80-nt sequences using RNAfold software^4^.

Two computational target prediction algorithms (TargetScan and miRanda) were used to predict the target genes of miRNA from blast matching against the *S. aequifasciatus* mRNA transcriptome sequence of this study. miRanda was used to match the entire miRNA sequences. The TargetScan (Shi et al., 2017) parameters were set as a context score percentile >50. The miRanda parameters (John et al., 2004) were set as free energy <−10 kcal/mol and a score >50. All miRNA targets were categorized into functional classes using the GO terms and KEGG pathway. And the results predicted by the two algorithms were combined, and the overlaps were calculated.

### Expression Analysis of mRNAs and miRNAs

mRNA expression level between different mRNA transcriptome were measured by RPKM (reads per kb per million reads) using RSEM software (Li et al., 2010; Li and Dewey, 2011). All transcript sequences obtained by splicing in Trinity were used as reference sequences. Each sample’s valid data alignment was quantified to the reference sequence. The main parameters were as follows: no-mixed, no-discordant gbar 1000, and end-to-end-k200. The mRNAs with log-fold difference ratios (log_2_ ratio) ≥1 and false discovery rate (<0.05) were considered to be significantly differentially expressed.

MicroRNA differential expression based on normalized deep-sequencing counts was analyzed by *t* test. Comparisons between testes and ovaries were made to identify significantly DEMs [|log_2_(fold change)| >1 and *P* ≤ 0.05]. Data normalization followed the procedures as described in a previous study (Cer et al., 2014).

### Quantitative Real-Time PCR Validation for miRNA and mRNA Expression

To validate the RNA-Seq data, eight mRNAs and eight miRNAs were randomly selected from differentially expressed mRNAs and miRNAs, and specific primers for mRNAs, miRNAs, 18S rRNA, and 5S rRNA (Supplementary Table S1) were designed to quantify their expression levels between ovaries and testes using quantitative real-time PCR (qRT-PCR). Total RNAs were reverse transcribed to cDNAs using M-MLV Reverse Transcriptase (Promega), with an equal amount of mixed reverse primer of the Oligo(dT)_18_ (Takara) and random primer [hexadeoxyribonucleotide mixture; pd(N)6] (Takara) for the quantification of mRNAs, and stem-loop RT primer (Supplementary Table S1) for the quantification of miRNAs.

Quantitative RT-PCR was performed using PowerUp^TM^ SYBR^TM^ Green Master Mix (Thermo Fisher Scientific Inc., Rockford, IL, United States) on a CFX96 Touch^TM^ Real-Time PCR Detection System (Bio-Rad, Heracles, CA, United States). The 18s rRNA that was not differentially expressed between testes and ovaries was used as an internal control for mRNA expression levels, and the 5s rRNA without differential expression between testes and ovaries was used as the internal control for the normalization of miRNA expression levels. Quantitative RT-PCR reactions of 20 μL contained 10.0 μL SYBR Green Master Mix, 1.0 μL forward primer (10 μM), and 1.0 μL reverse primer (10 μM), 1.0 μL cDNA, and 7.0 μL DEPC H_2_O, and the amplification procedure was carried out at 95°C for 2 min, 40 cycles of 95°C for 10 s, and 60°C for 20 s followed by disassociation curve analysis. Samples were replicated two times for each run, and the average Ct value was used to calculate gene expression levels. The relative expression levels were determined using the 2^–ΔΔCt^ method. All of data are expressed as the mean ± SEM. Comparisons between the two groups were performed using Student *t* tests. *P* < 0.05 was considered to indicate significant difference. All primers are listed in Supplementary Table S1.

## Results

### *De novo* Assembly and Functional Annotation of mRNA Transcriptome

Six cDNA libraries from three testes and three ovaries were sequenced using Illumina HiSeq 4000 sequencing. A total of 437,621,708 reads were obtained from six cDNA libraries (Table 1). After quality filtering using the Trinity *de novo* assembly method, we obtained 50,082 non-redundant genes (unigenes) with an average length of 885 bp and 65,496 transcripts with an average length of 1,077 bp (Table 2). All of the raw mRNA transcriptome sequencing data have been submitted to the SRA database^5^.

**TABLE 1:** Summary of Illumina HiSeq 4000 sequence reads.

**Sample**	**Raw data reads**	**Valid data reads**	**Q20 percentage**	**Q30 percentage**	**GC percentage**
Testis_1	88,682,568	86,322,782	97.26	92.56	50.25
Testis_2	82,098,772	79,904,932	97.39	92.72	50.72
Testis_3	54,797,130	53,361,960	97.28	92.51	49.13
Ovary_1	69,045,830	66,995,128	96.93	91.72	50.51
Ovary_2	62,969,370	61,204,582	97.27	92.41	51.22
Ovary_3	80,028,038	77,880,556	97.38	92.70	49.97

**TABLE 2:** *De novo* assembly statistics of the discus fish transcriptomic sequences.

**Category**	**All**	**Median GC%**	**Mean GC%**	**Mean length**	**N50 of contigs**
Gene	50,082	47.80	47.28	885	1,540
Transcript	65,496	48.10	47.64	1,077	1,896

The conservative analysis of the non-redundant transcripts showed that the transcripts were mainly distributed in fish, such as *Larimichthys crocea* (19%), *Lates calcarifer* (15.03%), *Seriola dumerili* (10.23%), *Seriola lalandi* (4.08%), *Stegastes partitus* (4.83%), and *Mastacembelus armatus* (4.25%) (Supplementary Figure S1). To annotate and analyze the putative functional roles of unigenes, all unigenes were compared with the Swiss-Prot database, Pfam database, and NCBI non-redundant nucleotide sequences (Nr) in the priority order of GO, eukaryotic Orthologous Groups (KOG) database, and KEGG database. A total of 25,026 unigenes (24.51%) were annotated in the NR database, and 22,739 unigenes in the Swiss-Prot, whereas the other unannotated unigenes represent novel genes of unknown functions (Table 3). A structured and controlled vocabulary to describe gene products was obtained using GO and KOG analysis. A total of 20,176 unigenes (40.29%) were assigned to the GO database (Table 3). Three distinct GO categories were characterized with 10 molecular function groups, 15 cellular component groups, and 25 biological process groups (Supplementary Figure S2). A total of 21,311 (42.55%) unigenes were assigned to the KOG database and classified into 25 functional categories (Supplementary Figure S3). Kyoto Encyclopedia of Genes and Genomes analysis was performed to identify potential candidate unigenes in biological pathways. A total of 15,702 (31.35%) unigenes were assigned to the KEGG database (Table 3) and mapped onto 36 predicted pathways (Supplementary Figure S4).

**TABLE 3:** Annotation statistics of the discus fish transcriptome sequencing.

**Gene_number**	**Swiss-Prot**	**Pfam**	**Nr**	**GO**	**KOG**	**KEGG**
50,082	22,739	20,450	23,371	20,176	21,311	15,702
100%	45.40%	40.83%	46.67%	40.29%	42.55%	31.35%

### Differentially Expressed mRNAs

**FIGURE 1:**
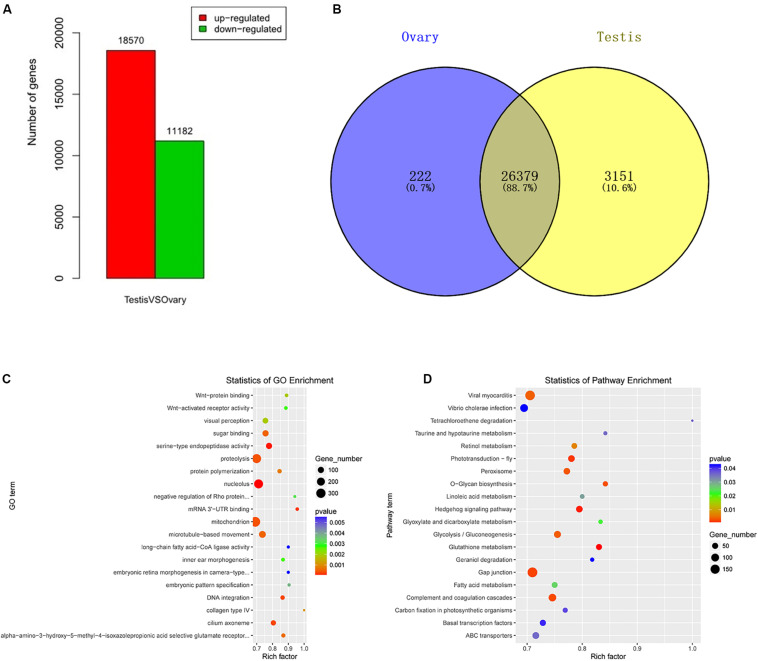
Identification, GO, and pathway of differentially expressed genes between testis and ovary. **(A)** Number of up-/down-expressed mRNAs in testis versus ovary. “Up-regulated” means that these unigenes were higher expressed in the testes comparing to the ovaries, and “down-regulated” means that these unigenes were lower expressed in the testes comparing to the ovaries; **(B)** the Venn diagram of testis-specific and ovarian-specific genes; **(C)** GO scatter diagram different expression genes between the testis and ovary; **(D)** KEGG scatter diagram different expression genes between the testis and ovary.

A total of 29,752 DEGs were detected by comparing DEGs between testis and ovary transcriptomic sequencing (Supplementary Table S2). A total of 18,570 of DEGs were higher expressed and 11,182 lower expressed in the testis compared to the ovary (Figure 1A). There were 3,151 genes that were specifically expressed in the testis, 222 in the ovary, and 26,379 in both tissues (Figure 1B). We can visually find the DEGs by a volcano plot (Supplementary Figure S5). The *x* axis shows the differences in expression given as the log values, whereas the *y* axis shows the significant differences in expression as negative log values. Differentially expressed genes are indicated by red dots, and non-DEGs are indicated by blue dots.

To further determine and compare the functions of these DEGs, a total of 122 GO terms including these DEGs were classified into three GO categories (cellular component, biological process, and molecular function) (Supplementary Table S3 and Figure 1C). The DEGs were compared to the KEGG pathway to gain an overview of the gene pathway networks. A total of 1,251 DEGs involved in 243 pathways were predicted in the pairwise comparison of testis versus ovary (*p* < 0.05) (Supplementary Table S4 and Figure 1D).

### Differentially Expressed miRNAs

**FIGURE 2:**
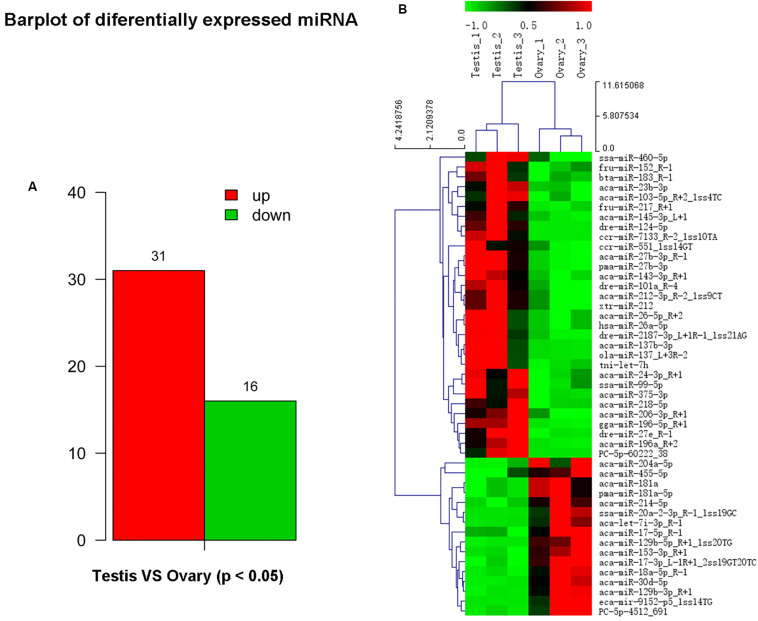
Differential expressed miRNAs between testis and ovary. **(A)** Number of up-/down-expressed miRNAs in testis and ovary. “Up” means that these miRNAs were higher expressed in the testes comparing to the ovaries, and “down” means that these miRNAs were lower expressed in the testes comparing to the ovaries; **(B)** heat map of the differentially expressed miRNAs in testis and ovary.

In this study, a total of 551 miRNAs were identified from the gonad tissues, and they ranged from 18 to 26 nt in length (Table 4). By comparing miRNA expression levels between testes and ovaries, a total of 47 DEMs were identified between testis and ovary (Supplementary Table S5). Thirty-one miRNAs were significantly higher expressed and 16 miRNAs significantly lower expressed in the testes comparing to the ovaries (Figure 2A and Supplementary Figure S6). We found that four miRNAs were specifically expressed in the testis, and they were dre-miR-124-5p, ccr-miR-7133, PC-5p-60222, and aca-miR-726, whereas three miRNAs were specifically expressed in the ovaries. Using the heat map of the DEMs, one can intuitively see the changes in miRNA expression (Figure 2B). These DEMs were regarded as candidate sex-related miRNAs.

**TABLE 4:** Distribution of these identified miRNAs in length.

**Length**	**Unique miRNA**	**Percentage**
18	24	4.36
19	35	6.35
20	35	6.35
21	93	16.88
22	266	48.28
23	76	13.79
24	13	2.36
25	6	1.09
26	3	0.54
All	551	100.00

### Integrated Analysis of DEMs and DEGs

**FIGURE 3:**
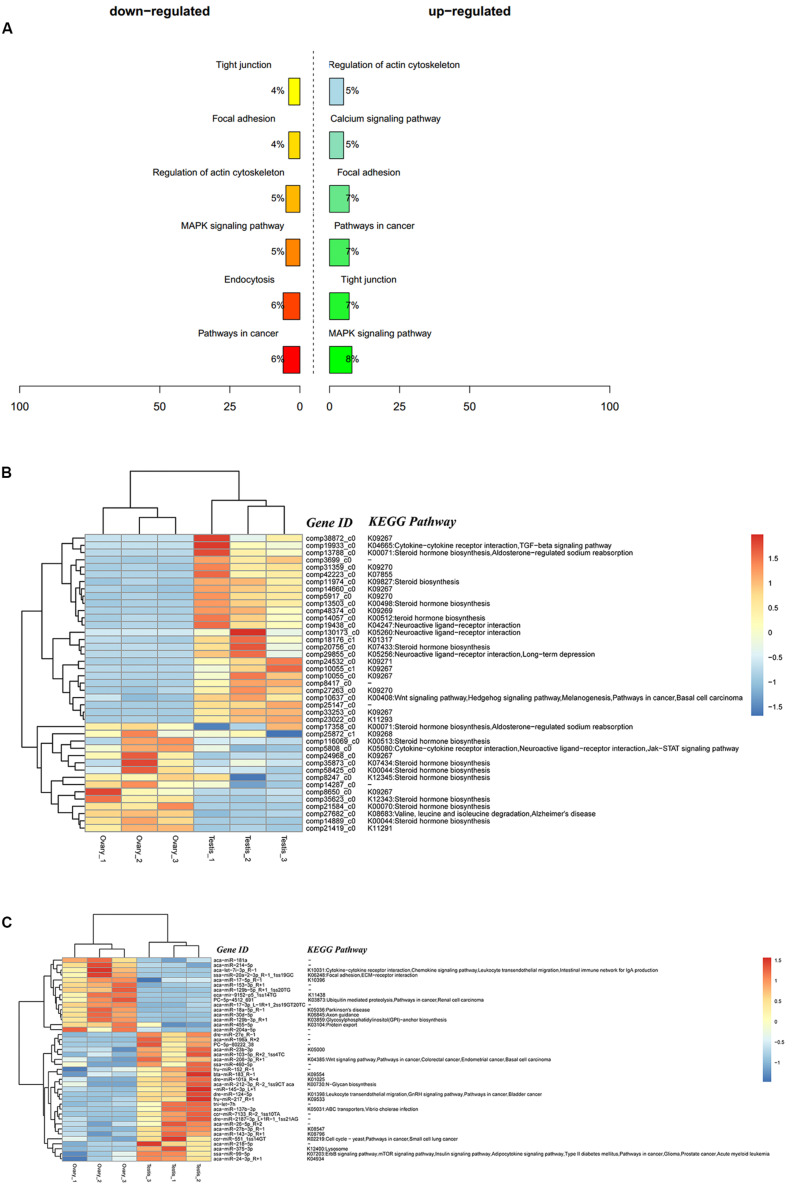
Integrated analysis results of DEMs and DEGs. **(A)** Percent of up-/down-expressed mRNAs associated with differentially expressed miRNAs in KEGG enrichment. “Up-regulated” means that these unigenes were higher expressed in the testes comparing to the ovaries, and “down-regulated” means that these unigenes were lower expressed in the testes comparing to the ovaries; **(B)** clustering and pathway distribution of 41 differentially expressed mRNAs; **(C)** clustering and pathway distribution of 41 differentially expressed miRNAs.

A total of 9,311 DEGs under the control of 47 miRNAs were identified, and of these, 5,600 were positively regulated, and 3,711 were negatively regulated. In the KEGG functional enrichment, by percentage, we identified the KEGG pathway that needs to be focused on in the joint analysis (Figure 3A). Approximately 41 DEGs were assigned to 37 signal pathways (Figure 3B), and DEMs were involved in 23 signal pathways (Figure 3C). We identified 41 miRNA–mRNA interaction pairs with 24 DEMs targeting 15 DEGs (Supplementary Table S7).

**FIGURE 4:**
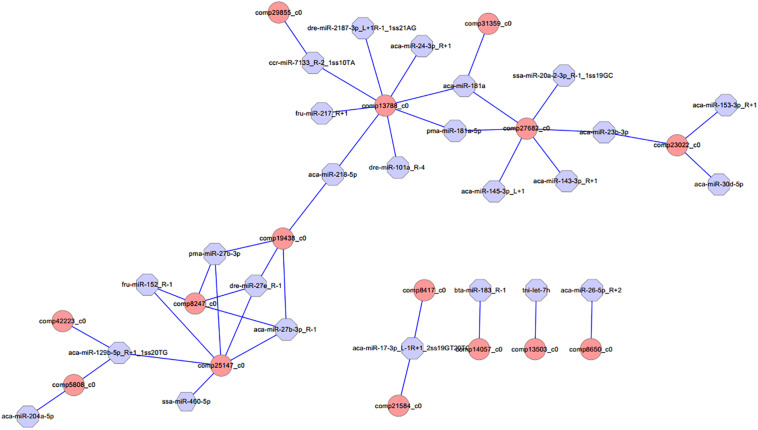
Proposed network of putative interactions between miRNAs and mRNAs in the gonad development. The regulation network of miRNAs and mRNAs involved in sexual development and maintenance is illustrated by Cytoscape. Pink represents miRNAs, and blue indicates their target genes.

Different expression of miRNAs with their predicted target genes was explored for cognate mRNA targets in their respective unigenes list in order to profile miRNA–mRNA functional interactions. These identified 41 miRNA–mRNA pairs were involved in sex differentiation and steroid hormone biosynthesis (Supplementary Table S7). Among these candidate miRNA–mRNA interaction pairs, each miRNA can target one or more genes, whereas each gene can be targeted by one or more miRNAs, which indicated that there were complex regulatory networks between miRNAs and gene mRNAs (Figure 4). For example, miR-2187-3p, miR-24-3p, miR-181a, miR-181-5p, miR-101a, miR-218–5p, miR-217, and miR-7133 can target *Hsd11*β*2*, whereas *Dmrt1* was targeted by miR-129-5p, miR-27b-3p, miR-27e, miR-152, miR-27b-3p, miR-17-3p, and miR-460.

### Validation of miRNA and mRNA Expression Using qRT-PCR

**FIGURE 5:**
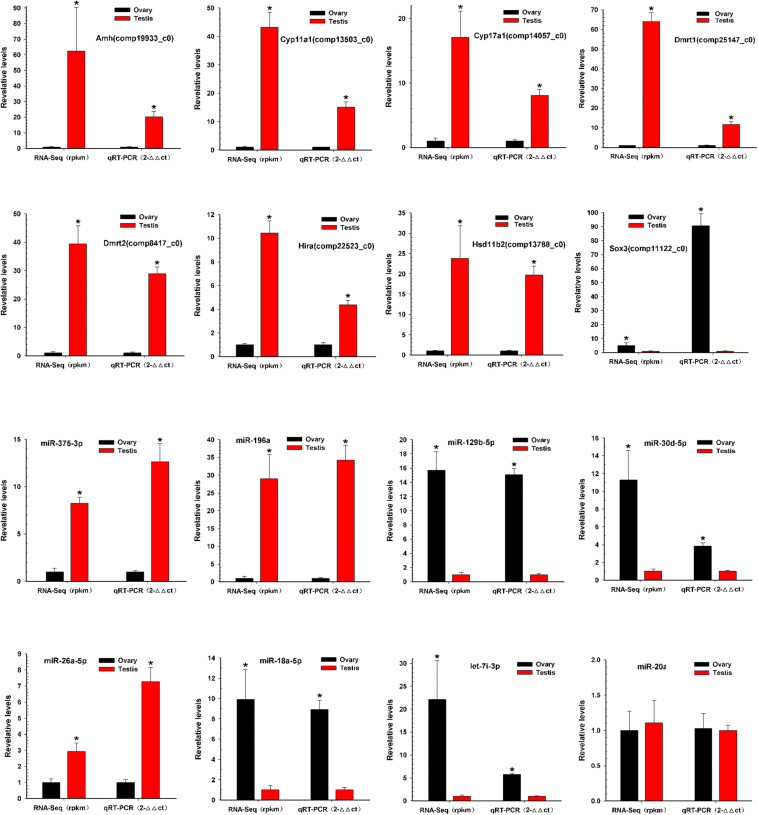
Validation of miRNAs and mRNAs expression between testis and ovary. Quantitative RT-PCR analysis of eight gene mRNAs and eight miRNAs selected randomly from RNA-Seq results between testis and ovary. All data were shown as mean ± SD. Student *t* test was used to identify significant differences. *P < 0.05 represents a statistically significant difference.

To verify the reliability and accuracy of the RNA-Seq results, we randomly selected eight genes and eight miRNAs to examine their expression levels between testis and ovary using qRT-PCR (Supplementary Table S6 and Figure 5). Expression results of genes indicated that *Amh*, *Cyp11a1*, *Cyp17a1*, *Dmrt1*, *Dmrt2*, *Hira*, and *Hsd11b2* were more highly expressed in the testis, whereas *Sox3* was more highly expressed in the ovary. Expression analysis of miRNAs indicated that miR-196a, miR-26a-5p, and miR-375-3p were up-regulated, and miR-129b-5p, miR-18a-5p, miR-30d-5p, and let-7i-3p were down-regulated. The qRT-PCR results for these eight miRNAs and eight mRNAs were similar to the RNA-Seq data.

## Discussion

In this study, using a high-throughput sequencing approach, we performed RNA-Seq analysis to study the expression of miRNAs and mRNAs in the gonads. The aim of the present work was to identify differentially expressed mRNAs and miRNAs between testes and ovaries and to predict miRNA possible targets, as well as to discover possible mechanisms responsible for sexual dimorphism in gonads.

In this study, *de novo* assembly revealed 50,082 non-redundant genes in the gonad. Based on the functional annotation of non-redundant genes, several sex-related metabolic pathways were summarized from other species. The main metabolic pathways of steroid hormone biosynthesis and oocyte meiosis were identified. Then, a total of 29,752 DEGs were identified between the ovary and testis tissue, including 18,570 up-regulated genes and 11,182 down-regulated genes in the testis compared with the ovary in the discus fish. Among these DEGs, some of the male-enhanced genes included the following the genes. *Dmrt1* (double-sex and mab-3–related transcription factor), which has been demonstrated to be the duplicated homologs of the medaka (*Oryzias latipes*). The *DMY* gene (Matsuda, 2003), which was first found as a sex-determination gene in non-mammalian vertebrates, has a conservative role in sex determination and differentiation of vertebrates as an ancestral function. *Dmrt2* gene may play a functional role in gonadal differentiation/development and germ cell maturation in the testis (Zhu et al., 2019). *Cyp17a1* is the qualitative regulator of steroidogenesis (Missaghian et al., 2009), and its expression level in the testis was higher compared to the ovary, which indicates that *CYP17a1* may be involved in testicular formation during sex differentiation (Sakurai et al., 2008). *Amh*, a male-specific gene, is derived from the Sertoli cells at the initiation of testis differentiation, participates in steroidogenic pathway to produce testosterone, or negatively controls estrogen production (Poonlaphdecha et al., 2011). Their expression levels were significantly higher in the testis than in the ovary using the RNA-Seq and qRT-PCR methods, which indicates that they may play key roles in regulating testis development in the discus fish.

Additionally, some of the female-enhanced genes were identified to be various ovary marker genes. These include the following several genes. *Sox3*, which is highly conserved in fish sex differentiation pathways (Takehana et al., 2014), plays a key role in regulating gametogenesis and gonad differentiation of vertebrates, and previous studies have shown that it might have more important effect in oogenesis than in spermatogenesis (Bo et al., 2007). *Cyp19a1*, a cytochrome P450 aromatase, which can catalyze the conversion of androgens to estrogens, plays dual roles in regulating testicular development during the initial period of sexual differentiation and later in ovarian development during the natural sex change in the protandrous black porgy (*Acanthopagrus schlegeli*) (Wu et al., 2008); In pejerrey (*Odontesthes bonariensis*), the tissue distribution analysis of *cyp19a1* mRNA in adult fish revealed high expression in the ovary, which is involved in the process of ovarian formation (Karube et al., 2007). *Dnd*, as an ovarian marker, plays an essential role during female gametogenesis and embryo development in pigs (Yang et al., 2012). However, *Sox9* expressed no difference between the testis and ovary in the discus fish. A possible reason is that *Sox9* participates in an early stage of gonadal development and is identified as an early signal of ovarian differentiation (Berbejillo et al., 2013).

We performed a comprehensive annotation and comparative analysis of miRNA using high-throughput sequencing and bioinformatics methods and identified 47 DEMs between the testis and the ovary in the discus fish, including 31 significantly up-regulated miRNAs and 16 significantly down-regulated miRNAs with testis compared to the ovary. In this study, the results from miRNA RNA-Seq sequencing indicated that miR-7641, miR-205a, miR-181a-5p, miR-143-3p, miR-145-3p, and miR-129-5p were differentially expressed between testis and ovary in the discus fish. MicroRNAs play crucial roles in a variety of biological processes via regulating expression of their target genes at the mRNA level. There is increasing evidence that miRNAs play an important role in regulating biological process, whereas miRNAs targeting mRNA are a key part of understanding their role in gene regulation networks (Jing et al., 2014; Zhang et al., 2016). In this study, 41 miRNA–mRNA interaction pairs were identified with 15 DEGs targeted by 24 DEMs.

MicroRNAs play crucial roles in a variety of biological processes via regulating expression of their target genes at the mRNA level. The miRNA–mRNA interaction pair with miRNA targeting mRNA is a key part of understanding miRNA function. In this study, 41 miRNA–mRNA interaction pairs were identified with 15 DEGs targeted by 24 DEMs using miRNA target prediction method. In these pairs, miR-2187-3p, miR-24-3p, miR-181a, miR-181-5p, miR-101a, miR-218–5p, miR-217, and miR-7133 can target *Hsd11*β*2*, which is involved in the steroid hormone biosynthesis pathway, indicating that these miRNAs might play an important role in regulating steroid hormone biosynthesis; miR-181a can also target *Hsd17*β*10*, which is involved in the steroid hormone biosynthesis pathway, and both were down-regulated in testes, indicating that miR-181a might exert different functions on regulating steroid hormone biosynthesis between ovaries and testes. Otherwise, previous studies showed that *Dmrt1* was predominantly expressed in spermatogonia, spermatocytes, and spermatids, as well as in Sertoli cells, indicating that *Dmrt1* plays an important role in spermatogenesis in *Halobatrachus didactylus* (Ubeda-Manzanaro et al., 2014). In the discus fish, *Dmrt1* was mainly expressed in the testes, whereas it was weakly expressed in the ovaries, and the *Dmrt1* gene was targeted by miR-129-5p, miR-27b-3p, miR-27e, miR-152, miR-27b-3p, miR-17-3p, and miR-460 in our miRNA target prediction data, which indicates that these might be involved in regulating spermatogenesis and testis development of the discus fish. Additionally, miR-129-5p was highly expressed in the ovary, was weakly expressed in the testis, and could target *Dmrt1*, GHR (growth hormone receptor), and RERG (ras-related and estrogen-regulated growth inhibitor), indicating that miR-129-5p might play important roles in regulating growth development and physiological function of the ovary in the discus fish. Previous studies have shown that *zp3* is found on the extracellular matrix of oocytes and acts as a receptor for mammalian sperm binding. In this study, we found that *zp3* can be targeted by miR-7641 and miR-205a in the miRNAs target prediction data, and miR-7641 and miR-205a were lowly expressed in the ovaries of the discus fish, whereas they were highly expressed in the testes, which indicates that lower expression of the two miRNAs in the ovaries might be necessary for expression of the *zp3* gene, playing an important role in regulating the biological process of the ovary binding sperm. Therefore, in the discus fish, miRNAs can play important roles in regulating steroid hormone synthesis, gonadal physiological function and sex development by regulating their target genes. However, the real function of these miRNAs needs to be confirmed in the gonad of the discus fish in the further studies.

## Conclusion

In our study, we found some DEGs and miRNAs between the ovary and testis and predicted target relations between genes and miRNAs using miRNA target prediction methods, and these DEGs and miRNAs were involved in regulating gonadal development, gametogenesis, and physiological function of ovary and testis. These results can help us further understand the mechanism of gonad development between female and male discus fish and provide data for further study of the sex development of the discus fish in the future.

## Data Availability Statement

The datasets generated for this study can be found in the Sequence Read Archive database (SRP148426).

## Ethics Statement

The animal study was reviewed and approved by Laboratory animal—Guideline for ethical review of Animal Welfare of China (GB/T 35892-2018) and Animal Ethics Committee of Shanghai Ocean University (SHOU-DW-2017-039).

## Author Contributions

ZC and JG designed the study. YF and ZX performed the bioinformatics analysis and verification experiments, and wrote the manuscript. BW provided important suggestions about the manuscript writing and collected the samples. All authors reviewed the manuscript.

## Conflict of Interest

The authors declare that the research was conducted in the absence of any commercial or financial relationships that could be construed as a potential conflict of interest.

## Footnotes

1 http://www.mirbase.org/cgi-bin/browse.pl

2 http://trinityrnaseq.sourceforge.net/

3 ftp://ftp.ncbi.nlm.nih.gov/blast/db/FASTA/nr.gz

4 http://rna.tbi.univie.ac.at/cgi-bin/RNAfold.cgi

5 https://www.ncbi.nlm.nih.gov/sra/SRP148426

## Supplementary Material

FIGURE S1 Conservative analysis of the non-redundant transcripts between species by comparing to the NR database.

FIGURE S2 Functional classification of assembled unique sequences based on gene ontology (GO) terms: molecular function, cellular component, and biological process.

FIGURE S3 Functional categories of KOG database in discus fish.

FIGURE S4 KEGG Pathway Classification of the gonad in discus fish.

FIGURE S5 Volcano plot of different expression genes between testis and ovary.

FIGURE S6 Venn diagram of different expression genes between testis and ovary. The 31 and 16 showed that they were expressed highly in testis and ovary, respectively.

TABLE S1 miRNA and mRNA Primers used in this study for qRT-PCR validation.

TABLE S2 The differential expression mRNA between testis and ovary.

TABLE S3 GO functional annotation of DEGs for testis and ovary.

TABLE S4 KEGG enrichment annotation of DEGs for testis and ovary.

TABLE S5 The differential expression miRNA between testis and ovary.

TABLE S6 The data for qRT-PCR and RNA-Seq of mRNAs and miRNA.

TABLE S7 41 miRNA-mRNA interaction pairs involved in sex development and maintenance between testis and ovary comparisons, respectively.

## Abbreviations

ANOVA: one-way analysis of variance
DEGs: differentially expressed genes
GO: Gene Ontology
KEGG: Kyoto Encyclopedia of Genes and Genomes
KOG: eukaryotic Orthologous Groups
MiRNAs: MicroRNAs
MS-222: Tricaine methanesulfonate
nt: nucleotides
qRT-PCR: quantitative real time PCR
RPKM: reads per kb per million reads
unigenes: non-redundant genes.
