# Supplementary figures and images for: Gonad-Specific Transcriptomes Reveal Differential Expression of Gene and miRNA Between Male and Female of the Discus Fish (Symphysodon aequifasciatus)
Source: Front Physiol. 2020 Aug 11;11:754. doi: 10.3389/fphys.2020.00754 (PMC7431700; doi:10.3389/fphys.2020.00754)

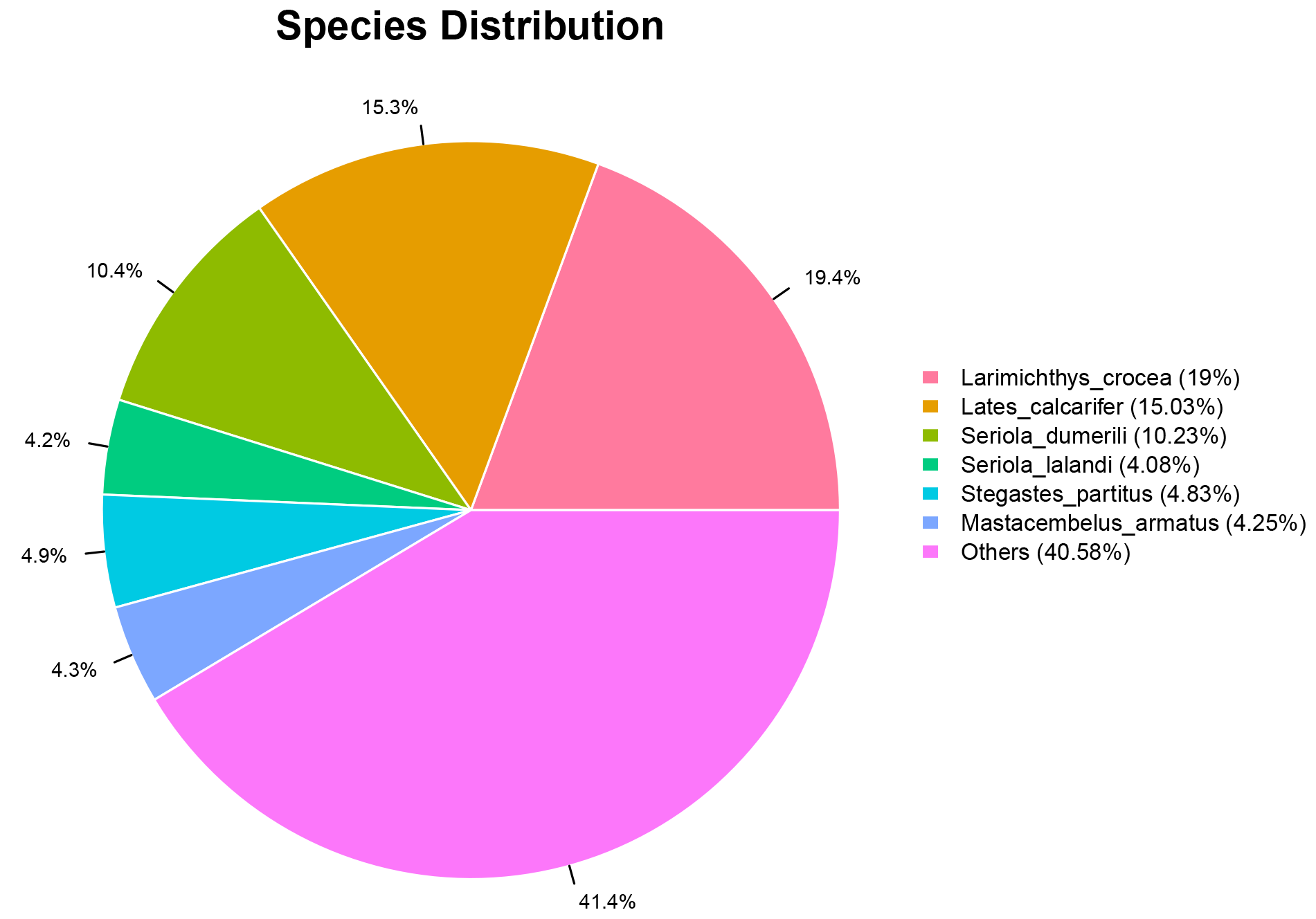

Supplement: FIGURE S1 — Conservative analysis of the non-redundant transcripts between species by comparing to the NR database. [file Image_1.tif]

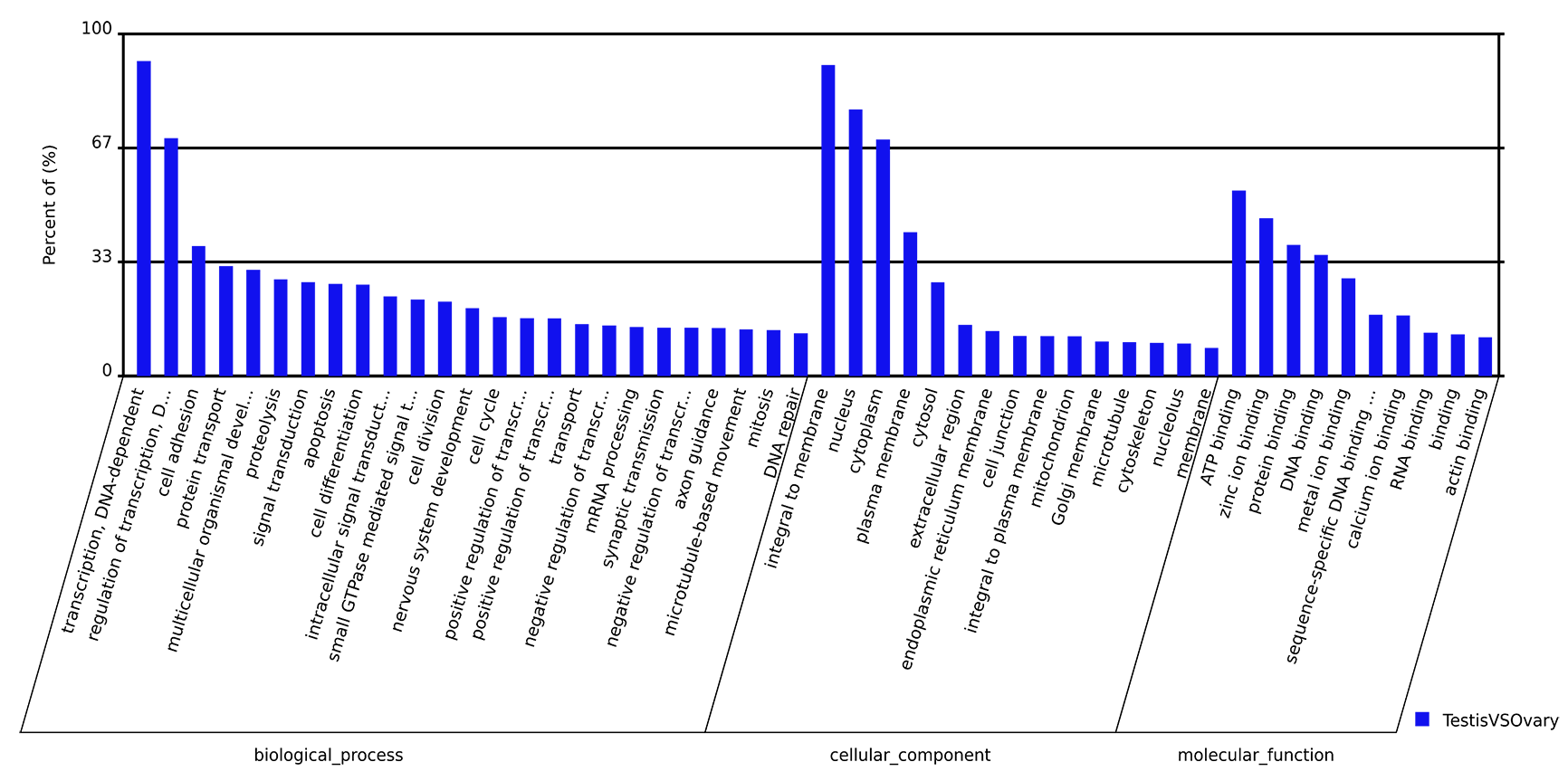

Supplement: FIGURE S2 — Functional classification of assembled unique sequences based on gene ontology (GO) terms: molecular function, cellular component, and biological process. [file Image_2.tif]

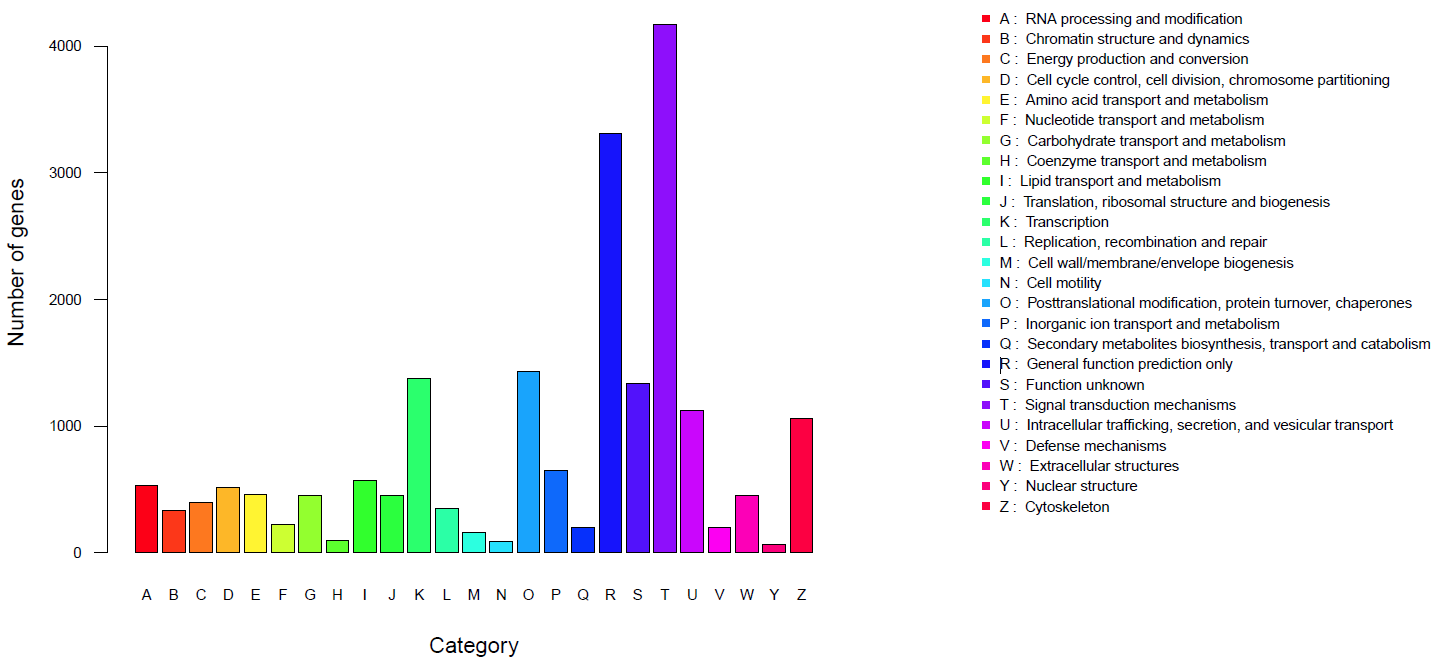

Supplement: FIGURE S3 — Functional categories of KOG database in discus fish. [file Image_3.tif]

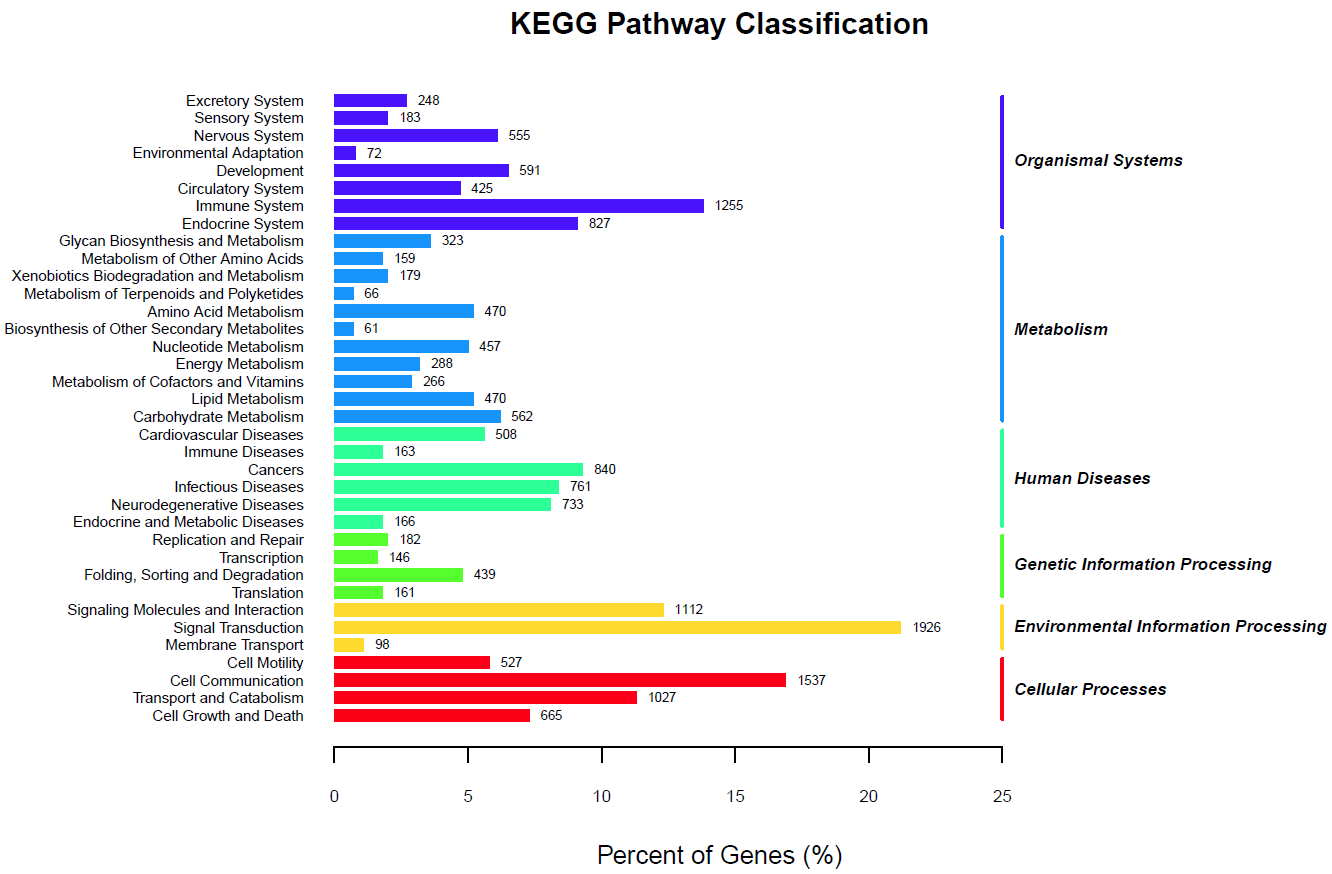

Supplement: FIGURE S4 — KEGG Pathway Classification of the gonad in discus fish. [file Image_4.tif]

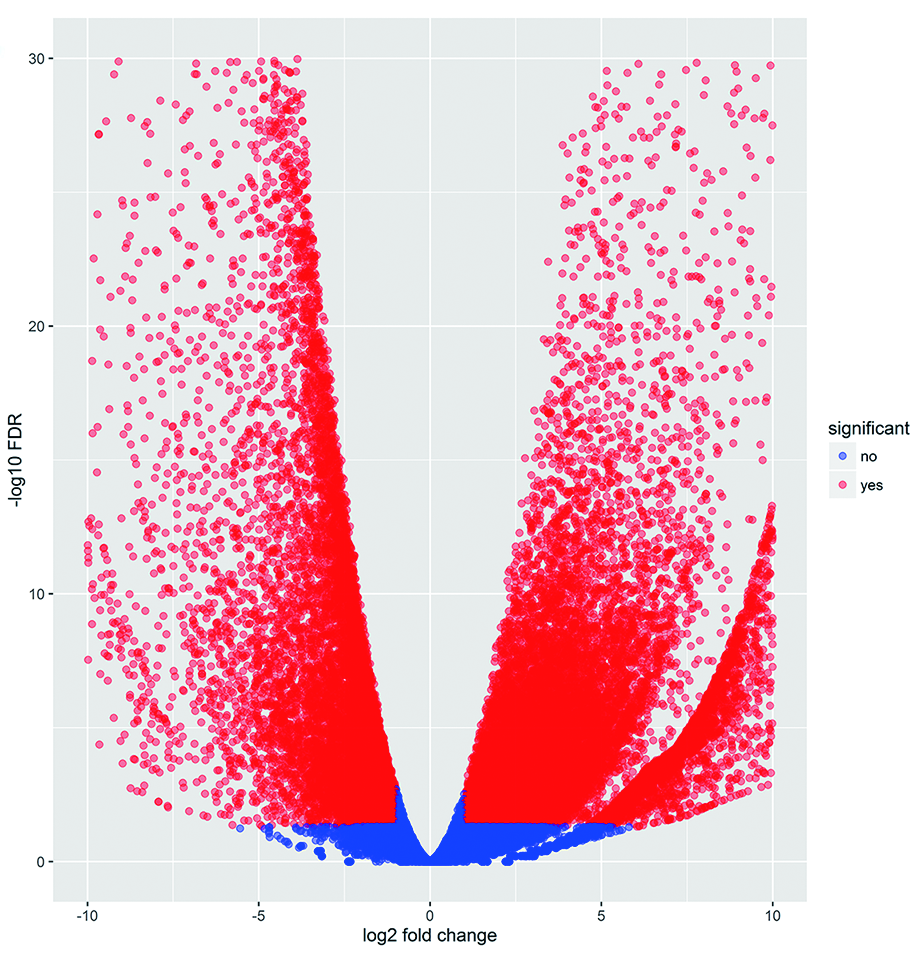

Supplement: FIGURE S5 — Volcano plot of different expression genes between testis and ovary. [file Image_5.tif]

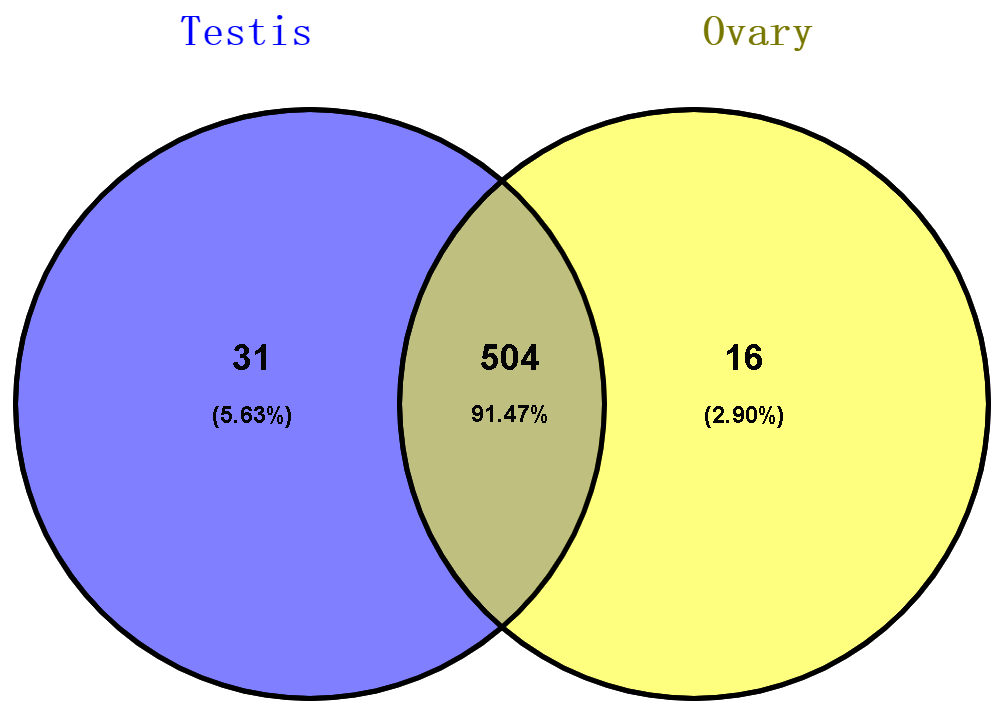

Supplement: FIGURE S6 — Venn diagram of different expression genes between testis and ovary. The 31 and 16 showed that they were expressed highly in testis and ovary, respectively. [file Image_6.tif]
